# Career commitment and professional satisfaction among Romanian pharmacy graduates: a nationwide cross-sectional survey

**DOI:** 10.3389/jpps.2026.15679

**Published:** 2026-03-05

**Authors:** Marius Călin Cherecheş, Aura Rusu

**Affiliations:** Faculty of Pharmacy, “George Emil Palade” University of Medicine, Pharmacy Science and Technology, Târgu Mureş, Romania

**Keywords:** career commitment, job satisfaction, pharmacy graduates, professional satisfaction, workforce retention

## Abstract

**Introduction:**

Professional satisfaction is a key determinant of career commitment and workforce sustainability and retention in the pharmacy sector. The study examines professional satisfaction among Romanian pharmacy graduates by analysing hygiene factors, intrinsic motivators, and perceptions of pay equity, assessing sector-based differences and exploring associations between these dimensions and long-term career commitment.

**Methods:**

A cross-sectional survey was conducted among 473 pharmacy graduates (2009–2023). Professional satisfaction was evaluated using 13 structured items from a questionnaire that covered hygiene factors, intrinsic motivators, and perceptions of pay equity. Reliability was assessed with Cronbach’s α. Descriptive statistics, ANOVA, and chi-square tests were applied.

**Results:**

The Hygiene Index (α = 0.67; M = 3.28, SD = 0.76) and Motivators Index (α = 0.80; M = 3.22, SD = 0.86) reflected moderate satisfaction. The Pay-Equity Index showed very low scores (α = 0.77; M = 1.86, SD = 0.70). Salary satisfaction (M = 2.22, SD = 1.23) and expectations for future salary increases (M = 2.21, SD = 1.06) were rated as the lowest. Over 80% perceived their income as “much lower” than that of physicians or dentists. Only 7% stated they would “definitely” choose pharmacy again, while 46% responded “definitely not,” and over 70% expressed some degree of non-recommitment. Community pharmacists consistently reported lower satisfaction across indices compared to peers in industry or education.

**Conclusion:**

Romanian pharmacists report moderate satisfaction with work conditions and collegiality, but widespread dissatisfaction with pay equity and career opportunities. Alarmingly, almost three-quarters of pharmacists said they would not choose pharmacy again, indicating a lack of professional commitment. The results raise substantial concerns about professional commitment and suggest a risk to the long-term sustainability of the Romanian pharmacy workforce. Urgent policy interventions are needed to address salary disparities, improve recognition, and expand career development pathways to retain qualified professionals and ensure the resilience of pharmaceutical services.

## Introduction

Professional satisfaction is increasingly recognized as a fundamental element of healthcare workforce sustainability, influencing retention, service quality, and career longevity. In this study, long-term engagement means sustained professional commitment to pharmacy, shown by willingness to re-choose pharmacy education if given the chance. It reflects graduates’ retrospective evaluation of their career and indicates enduring attachment to the profession. In the pharmacy sector, where graduates pursue diverse career paths (from community and hospital settings to industry and academia), understanding the factors that shape long-term engagement is critical. Despite global attention to pharmacist satisfaction, Eastern European contexts remain underexplored, particularly Romania, where systemic challenges such as low pay, limited advancement, and institutional under-support persist.

The pharmaceutical sector faces a considerable challenge due to the diverse career pathways accessible to graduates, including community and hospital pharmacy, the pharmaceutical industry, academia, and regulatory agencies [1, 2]. Studies indicate that professional satisfaction is not only predictive of long-term retention but also influences pharmacists’ relationships with patients, levels of trust, and perceived quality of services [3, 4]. Pharmacist satisfaction has been recognised as a critical factor in determining the resilience and sustainability of health systems worldwide, particularly in light of the increased mobility and structural pressures on the healthcare workforce [5].

Theoretical frameworks offer valuable insights into comprehending the multifaceted aspects of professional satisfaction. Herzberg’s Two-Factor Theory differentiates between hygiene factors—external elements such as working conditions, salary, and job security—and motivators, which encompass internal drivers like recognition, achievement, and advancement opportunities [6–8]. Studies in pharmacy environments validate this differentiation: whereas poor working conditions or insufficient compensation swiftly lead to discontent, genuine engagement and career commitment are chiefly influenced by factors such as autonomy, acknowledgement, and professional advancement [9, 10]. Adams’ Equity Theory also emphasises the importance of feeling that things are fair, particularly in terms of pay. Comparative salary evaluations between pharmacists and other healthcare professionals, including doctors and dentists, often serve as benchmarks for perceived equity, influencing job satisfaction and retention [11, 12].

But hygiene and fairness issues are only part of the story. Research indicates that work-life quality, supportive workplace relationships, and institutional endorsement of continuing education are equally vital for maintaining motivation and mitigating burnout [13–15]. Evidence from community and hospital pharmacies globally indicates that while hygiene factors mitigate dissatisfaction, they do not ensure engagement; intrinsic motivators are the fundamental determinants of long-term career commitment [16–18]. Nonetheless, ongoing difficulties such as increased workloads, constrained career opportunities, and unequal compensation have been linked to elevated stress levels and attrition rates, especially among early-career pharmacists [19]. A considerable amount of current research focuses on specific facets of satisfaction—such as burnout, compensation, or work-life balance—without integrating them into a holistic multidimensional framework [20].

The body of evidence concerning pharmacists’ professional satisfaction in Eastern Europe is growing, but it remains poorly organised. Bulgarian pharmacists experience significant stress and moderate job satisfaction, indicating systemic problems in working conditions and compensation [21]. Recent studies in Romania indicate a complex environment characterised by tension between extrinsic and intrinsic factors. Surveys and qualitative analyses show ongoing dissatisfaction with salaries and institutional support, while also highlighting the motivational importance of recognition and patient-centred practice [22]. Netnographic studies reveal the daily frustrations and coping strategies of Romanian pharmacists, emphasising the interplay between structural constraints and human resilience. [23]. Furthermore, studies on leadership and professional development emphasise the need for more robust institutional support systems to sustain motivation and enhance career opportunities [24].

Previous analyses by authors investigated the economic, demographic, and legislative determinants affecting the expansion of pharmacies in Romania, demonstrating how market dynamics and policy decisions create systemic pressures that invariably influence working conditions and levels of satisfaction [25].

Although these advancements have been made, considerable deficiencies in our understanding remain. In Romania, there is a limited number of quantitative studies that systematically assess satisfaction across hygiene, motivator, and equity dimensions using validated theoretical frameworks. Due to the rapid transformations in Romanian pharmacy labour markets and education needs over the past 15 years, there is an immediate need for data that covers both extrinsic and intrinsic factors influencing satisfaction. Given the growing concerns about pharmacist retention and healthcare system resilience, understanding the drivers of career dissatisfaction is essential for informing national workforce policies.

This study aims to address these shortcomings by examining professional satisfaction among Romanian pharmacy graduates through three interconnected frameworks: Herzberg’s hygiene-motivator theory, Adams’ equity theory, and Adams’ equity-based perceptions of compensation. Utilizing data from Section *Conclusion* of a nationwide survey (items 5.1–5.13), we construct composite indices for each dimension and assess their reliability. Our goal is to identify the primary determinants affecting satisfaction, their relative importance, and their relationship with career commitment, defined as graduates’ willingness to pursue a career in pharmacy again. This comprehensive paradigm offers new empirical insights into the literature on pharmacy workforce satisfaction in Romania and provides a basis for comparison with global trends.

## Methods

### Study design and data collection

This study employed a cross-sectional survey design targeting pharmacy graduates from Romanian universities who completed their studies between 2009 and 2023. From April 2024 to August 2025, a structured online questionnaire was used to gather data via Google Forms. The survey link was shared on social media, through alumni networks, and with professional associations to reach a broad audience nationwide. Participants included graduates from all university institutions in Romania. The recruitment relied on voluntary participation and convenience sampling via professional networks and social media, which may introduce self-selection bias by overrepresenting individuals with strong experiences. Thus, findings should be cautiously interpreted for national generalization.

The current article is based on the same dataset as our previous study on educational satisfaction and curricular relevance, which analysed survey Sections *Introduction, Methods, Results, Discussion*. For this analysis, we focus specifically on Section *Conclusion*, which evaluated professional satisfaction, while including demographic variables for subgroup comparisons. While the previous publication analyzed educational satisfaction and curricular relevance (Sections *Introduction, Methods, Results, Discussion* of the survey), the present study focuses exclusively on professional satisfaction (Section *Conclusion*), employing a distinct theoretical framework grounded in Herzberg’s motivation theory and Adams’ equity theory. The analytical objectives and constructed indices therefore differ substantially between the two studies.

### Questionnaire development

The questionnaire was designed to operationalise three complementary theoretical models of motivation and satisfaction:

- Herzberg’s Two-Factor Theory: Distinguishing hygiene factors (extrinsic conditions, such as work environment, salary, job security, and colleague relationships) from motivators (intrinsic factors, including career prospects, support for professional development, recognition, fulfilment, and work challenge).
- Equity Theory (Adams): Examining perceived fairness in remuneration through income comparisons with relevant professional groups (medical doctors, dentists, and peers with similar academic training).
- Career Commitment: Assessed through respondents’ willingness to re-choose pharmacy education if given the opportunity, reflecting long-term professional attachment.

The items included in Section *Conclusion* were developed by the research team based on established theoretical constructs derived from Herzberg’s Two-Factor Theory and Adams’ Equity Theory, as well as relevant literature on healthcare workforce satisfaction. Although the instrument was not directly adapted from a single previously validated scale, internal consistency testing in the present sample supported its reliability.

Professional satisfaction was measured using 13 items (5.1–5.13) from Section *Conclusion* of the questionnaire. Items were presented on five-point Likert-type scales, with higher scores consistently coded to indicate greater satisfaction.

Three composite indices were constructed:

- Hygiene Index (4 items): Work conditions, colleague relationships, salary satisfaction, and job security (Items 5.1, 5.2, 5.3, 5.6).
- Motivators Index (5 items): Career prospects, support for professional development, personal fulfilment, recognition frequency, and work challenge (Items 5.8–5.12).
- Pay-Equity Index (3 items): Income comparisons with medical doctors, dentists, and similarly educated peers (Items 5.7a–c).

The outcome variable was the intention to re-choose pharmacy education (Item 5.13). The item on income range (Item 5.4) and expectations for salary increase (Item 5.5) were treated descriptively and not included in reliability testing or composite indices. Data cleaning procedures involved recoding inconsistent entries to ensure a uniform 1–5 scale across items.

At the beginning of the online questionnaire, participants were provided with an information statement describing the study purpose, voluntary nature of participation, and confidentiality assurances. As indicated in the introductory section of the form, continuation and completion of the questionnaire were considered to imply informed consent. No personally identifiable information was collected. The dataset was stored locally by the principal investigator and used exclusively for research purposes.

### Statistical analysis

Data analysis was conducted using Stata/SE 16.0 [26]. Descriptive statistics (means, standard deviations, frequencies) were calculated for all items and indices. Internal consistency was assessed using Cronbach’s α for the entire scale (items 5.1–5.12) and for each composite index separately. We used one-way ANOVA to test differences in satisfaction indices (Hygiene, Motivators, Pay-Equity) across employment categories (community, hospital, industry, academic, regulatory). Bonferroni post-hoc tests were applied to adjust for multiple comparisons. Associations between categorical outcomes (e.g., willingness to re-choose pharmacy) and demographic/professional variables (graduation year, university, age group, sector) were examined using Pearson’s chi-square tests. Statistical significance was set at *p* < 0.05.

### Ethical approval

Ethical approval was obtained from the Institutional Review Board of “George Emil Palade” University of Medicine, Pharmacy, Science, and Technology of Târgu Mureş (approval no. 3049, April 12, 2024). Participation was voluntary, and responses were anonymous and confidential.

## Results

### Sample characteristics and reliability analysis

The analytical sample comprised 473 pharmacists who graduated between 2009 and 2023 and completed the survey.

Participants represented all 11 accredited pharmacy faculties in Romania. The largest proportion graduated from UMFST G.E. Palade Târgu Mureş (39.53%), followed by UMF Carol Davila Bucureşti (16.91%) and UMF Iuliu Hațieganu Cluj-Napoca (13.11%). The remaining respondents were distributed across UMF Gr. T. Popa Iaşi, UMF Victor Babeş Timişoara, UMF Craiova, Universitatea Oradea, Universitatea Dunărea de Jos Galați, Universitatea Ovidius Constanța, Universitatea Vasile Goldiş Arad, and Universitatea Titu Maiorescu Bucureşti.

The largest percentage of graduates were working in community pharmacy (n = 325; 68.7%), followed by the pharmaceutical industry (n = 57; 12.1%), other professional sectors (n = 42; 8.9%), training or residency jobs (n = 33; 7.0%), and the public sector/education (n = 16; 3.4%).

Participants presented a diverse variety of demographic features, predominantly concentrated in the 28–35 category (52.2%), representing both early-career and mid-career professionals. The sample was primarily female (88.4%), reflecting the well-established predominance of women in Romanian pharmacy education and professional practice. This distribution highlights the sample’s representativeness in reflecting the predominant occupational trajectories and demographic trends of pharmacy graduates post-2009, as illustrated in Table 1.

**TABLE 1:** Demographic and employment characteristics of respondents.

Characteristic	Categories	n	%
Gender	Female	418	88.4
	Male	55	11.6
Age group	20–27	125	26.4
	28–35	247	52.2
	36–45	78	16.5
	46–65	23	4.9
Sector of employment	Community pharmacy	325	68.7
	Pharmaceutical industry	57	12.1
	Other sectors	42	8.9
	Training/residency	33	7.0
	Public sector/education	16	3.4

Before turning to descriptive findings, we assessed the internal consistency of the survey items. Cronbach’s α for the combined set of items 5.1–5.12 (excluding item 5.4, which pertains to the income range) was 0.82, indicating good internal reliability for the overall scale; this suggests that the items collectively measure a coherent construct of professional satisfaction.

At the level of composite indices, reliability was also satisfactory. The Hygiene Index (items 5.1, 5.2, 5.3, 5.6) had an α of 0.67, representing an acceptable level for exploratory research. The Motivators Index (items 5.8–5.12) achieved α = 0.80, demonstrating strong consistency, while the Pay-Equity Index (items 5.7a–c) reached α = 0.77, also within the acceptable-to-good range. Together, these coefficients confirm that the indices can be reliably used in subsequent analyses to capture latent dimensions of professional satisfaction. All data are presented in Table 2.

**TABLE 2:** Reliability and descriptive statistics for composite indices.

Index	Items	Cronbach’s α	Mean	SD
Hygiene	5.1, 5.2, 5.3, 5.6	0.67	3.28	0.76
Motivators	5.8–5.12	0.80	3.22	0.86
Pay-equity	5.7a–c	0.77	1.86	0.70
Full scale (5.1–5.12)	12 items	0.82	—	—

### Item-level descriptive statistics

Table 3 shows descriptive data for items in Section *Conclusion*. Hygiene variables, such as relationships with colleagues and job security, received moderate to good ratings. However, wage satisfaction (Mean = 2.22, SD = 1.23) and salary expectations (Mean = 2.21, SD = 1.06) scored the lowest. Income comparisons revealed significant perceived differences, with 81% feeling that their income was significantly lower than that of physicians’ and 79% lower than that of dentists’. Motivational factors (e.g., fulfilment, challenge) got reasonable scores, but recognition was low (Mean = 2.77, SD = 1.08). The willingness to re-select pharmacy education (item 5.13) was low (Mean = 2.04, SD = 1.26), with 46% saying “definitely not.”

**TABLE 3:** Item-level descriptive statistics (Section *Conclusion*).

Item	Label	N	Mean	SD	Min	Max	Cat 1 (%)	Cat 2 (%)	Cat 3 (%)	Cat 4 (%)	Cat 5 (%)	Cat 6 (%)
5.1	Work conditions	473	3.55	1.05	1	5	4.44	12.47	23.89	42.49	16.70	—
5.2	Relations with colleagues	468	3.75	1.00	1	5	2.35	10.04	21.37	42.95	23.29	—
5.3	Salary satisfaction	473	2.22	1.23	1	5	38.69	24.52	16.91	15.64	4.23	—
5.4	Monthly income range^a^	473	2.62	0.92	1	6	2.75	50.74	34.88	6.98	2.33	2.33
5.5	Expected salary increase	426	2.21	1.06	1	5	30.99	33.10	21.83	12.44	1.64	—
5.6	Job security	473	3.62	0.98	1	5	2.96	9.94	26.85	43.13	17.12	—
5.7a	Income vs. doctors	473	1.32	0.81	1	5	80.97	12.26	3.17	0.85	2.75	—
5.7b	Income vs. dentists	473	1.35	0.85	1	5	78.86	13.95	3.38	0.63	3.17	—
5.7c	Income vs. peers	473	2.89	0.88	1	5	6.55	20.30	54.97	13.53	4.65	—
5.8	Career prospects^b^	448	2.95	0.81	1	4	1.12	31.70	38.39	28.79	—	—
5.9	Support for professional development	469	3.14	1.17	1	5	11.09	15.99	34.33	25.37	13.22	—
5.10	Personal fulfilment	473	3.28	1.30	1	5	10.99	21.14	16.91	31.08	19.87	—
5.11	Recognition	473	2.77	1.08	1	5	12.90	29.18	30.44	23.04	4.44	—
5.12	Work challenge	473	3.37	1.09	1	5	7.19	13.32	27.27	39.53	12.68	—
5.13	Would re-choose pharmacy	468	2.04	1.26	1	5	45.94	29.27	7.05	10.68	7.05	—

Cat 1–Cat 6 refer to the percentage of respondents selecting each response category on the original Likert-type scale (range depending on the item: 1–5 or 1–6).

^a^ Item 5.4 has six ordered categories (1–6).

^b^ Item 5.8 is a 4-point scale; values for %5 and %6 not applicable.

Several significant trends emerge. Initially, reactions to the hygiene elements (items 5.1, 5.2, 5.6) show fairly positive attitudes. For example, interpersonal interactions with colleagues received the highest rating among hygiene factors (Mean = 3.75, SD = 1.00), with nearly two-thirds of participants rating them as “good” or “very good.” Work conditions (Mean = 3.55, SD = 1.05) and job security (Mean = 3.62, SD = 0.98) surpassed the neutral midpoint, indicating overall satisfaction in both areas. Conversely, ratings concerning salary were notably poor. Satisfaction with salary (item 5.3) received some of the lowest scores on the scale (Mean = 2.22, SD = 1.23), with over two-thirds of respondents choosing the two lowest categories (“very dissatisfied” or “dissatisfied”). Similarly, expectations for future income increases (item 5.5) were pessimistic, with an average of 2.21 (SD = 1.06), and nearly 64% of participants indicated “very unlikely” or “unlikely.”

Data about salary comparisons indicated significant perceived disparities. A significant 81% of respondents assessed their income as “much lower” than that of physicians (item 5.7a), while 79% made the same assessment in comparison to dentists (item 5.7b). In contrast, attitudes were relatively more equitable when compared to peers with similar qualifications (item 5.7c, Mean = 2.89, SD = 0.88), with a slight majority (55%) indicating they were “similar” to their colleagues.

The findings on the motivating elements varied. Personal fulfilment (item 5.10, Mean = 3.28, SD = 1.30) and job challenge (item 5.12, Mean = 3.37, SD = 1.09) received moderately positive scores, indicating that many respondents see some importance and mental stimulation in their work. However, the recognition of achievements (item 5.11) was notably lower (Mean = 2.77, SD = 1.08), with 42% of participants stating that recognition occurred “rarely” or “very rarely.” Support for professional development (item 5.9) remained modest (Mean = 3.14, SD = 1.17), reflecting a varied experience of institutional support.

The outcome item (5.13), which questioned if graduates would select pharmacy again, produced some of the most troubling results. The average response was 2.04 (SD = 1.26), with approximately 74% of respondents selecting options in the “definitely not,” “unlikely,” or “neutral” categories. Indeed, 46% unequivocally indicated “definitely not.” This distribution suggests considerable discontent with the profession choice among most graduates, despite moderate happiness with certain facets of work life.

### Composite indices

Following the item-level findings, we aggregated individual items into three composite indices, consistent with Herzberg’s paradigm and later changes in pharmacy workforce research. The Hygiene Index was derived from elements 5.1 (working environment), 5.2 (collegial connections), 5.3 (salary satisfaction), and 5.6 (job security). The Motivators Index included items 5.8 (career possibilities), 5.9 (support for professional advancement), 5.10 (personal fulfilment), 5.11 (recognition), and 5.12 (job challenge). The Pay-Equity Index was ultimately developed from questions 5.7a–c, which evaluated views of salary in comparison to physicians, dentists, and peers with equivalent qualifications. Item 5.4 (income range) was excluded from composite scores but is reported independently.

Table 2 presents the descriptive data for the indices. The Hygiene Index yielded a mean score of 3.28 (SD = 0.76), indicating moderate satisfaction with contextual elements, including the working environment and job security. The Motivators Index averaged 3.22 (SD = 0.86), suggesting a similar yet marginally more favourable inclination towards intrinsic variables, such as fulfilment and challenge. The Pay-Equity Index was significantly lower, at 1.86 (SD = 0.70), indicating widespread discontent with income equity among professionals.

**FIGURE 1:**
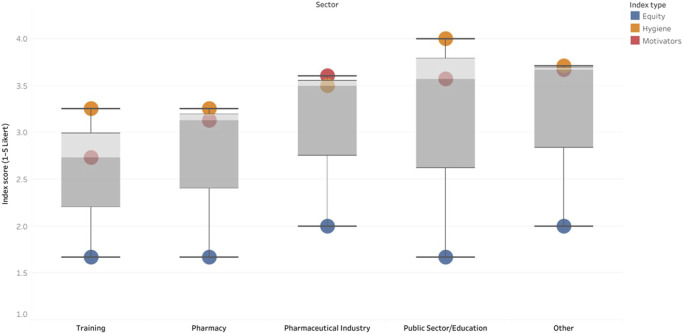
Distribution of professional satisfaction indices (Hygiene, Motivators, pay-equity) across employment sectors.

Figure 1 illustrates mean scores for each index across employment sectors. While the overall pattern of moderate hygiene and motivators, but low pay equity was consistent across the dataset, sectoral variation was evident. Community pharmacists reported the lowest scores across all indices. In contrast, respondents employed in the pharmaceutical industry and in the public sector/education displayed comparatively higher values, particularly for hygiene and motivators.

### Income and equity perceptions

Besides composite indices, we examined specific income-related factors to contextualise graduates’ financial evaluations. Table 4 displays descriptive statistics for the monthly income range (item 5.4) and expected salary increase (item 5.5). Fifty-one per cent of respondents reported monthly wages between 3,000 and 4,500 lei, while a further thirty-five per cent fell within the 4,501–7,000 lei group. A small number of respondents reported earnings exceeding 6,000 lei. These results highlight the narrow range of pharmacist incomes, primarily within Romania’s lower-to-middle income bracket.

**TABLE 4:** Descriptive statistics for income range and salary expectations.

Item	Categories	n	%	Mean	SD
Income range (5.4)	≤3,000 lei	13	2.8	2.62	0.92
	3,001–4,500 lei	240	50.7	—	—
	4,501–7,000 lei	165	34.9	—	—
	7,001–10,000 lei	33	7.0	—	—
	10,001–15,000 lei	11	2.3	—	—
	≥15,000 lei	11	2.3	—	—
Expected salary increase (5.5)	Very unlikely (1)	132	31.0	2.21	1.06
	Unlikely (2)	141	33.1	—	—
	Neutral (3)	93	21.8	—	—
	Likely (4)	53	12.4	—	—
	Very likely (5)	7	1.6	—	—

Expectations for future income growth were modest. Two-thirds of graduates indicated that significant wage increases were either very unlikely (31%) or unlikely (33%). Only 14% considered such increases probable or highly probable. The average score for this item was 2.21 (SD = 1.06), indicating a general lack of optimism regarding upward financial mobility.

**FIGURE 2:**
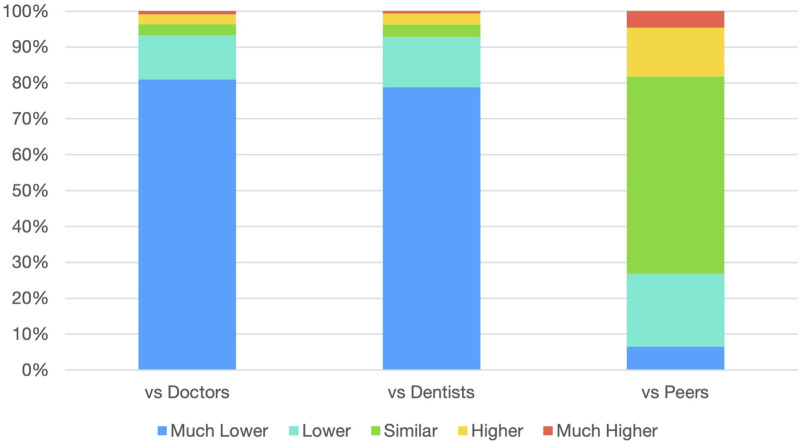
Income comparison perceptions among pharmacy graduates (n = 473).

Perceptions of income equity were much more significant. According to Table 1, 81% of respondents believed their earnings were considerably lower than those of physicians, while 79% felt the same about dentists. Comparisons with equally qualified peers (item 5.7c) were less negative, with 55% selecting the same group; however, the overall average remained below 3. The data show that although intra-professional comparisons provide some balance, inter-professional comparisons between physicians and dentists heavily influence perceptions of inequality Figure 2.

**FIGURE 3:**
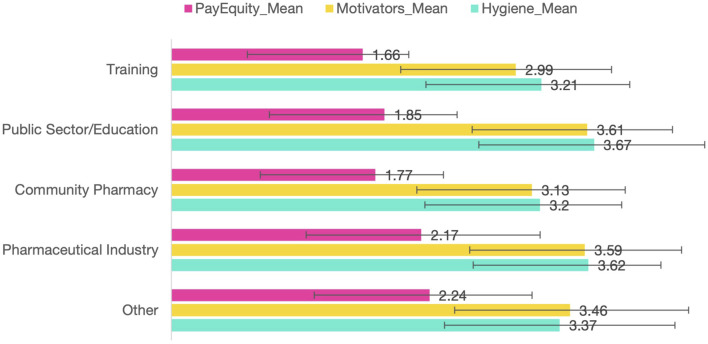
Sectoral differences in professional satisfaction indices (means ± SD, N = 473).

Figure 3 shows the detailed distribution of reported income ranges. Together, these figures underscore the dual challenge faced by graduates: objectively modest salaries and subjectively perceived inequities relative to benchmark professions.

### Sector differences

We conducted additional tests to examine variations in the composite indices among employment sectors utilising one-way analysis of variance (ANOVA). The results demonstrated statistically significant differences across all three indices: Hygiene (F(4,468) = 5.30, p < 0.001), Motivators (F(4,468) = 6.03, p < 0.001), and Pay-Equity (F(4,468) = 8.27, p < 0.001). The results are encapsulated in Table 5.

**TABLE 5:** ANOVA results for sectoral differences in composite indices.

Index	F(df)	*p*-value	The sector with the highest mean	The sector with the lowest mean
Hygiene	5.30 (4,468)	<0.001	Public sector/education	Community pharmacy
Motivators	6.03 (4,468)	<0.001	Pharmaceutical industry	Community pharmacy
Pay-equity	8.27 (4,468)	<0.001	Pharmaceutical industry	Community pharmacy

The results exhibited a consistent pattern across indices: graduates employed in community pharmacy reported markedly lower satisfaction than their counterparts in the pharmaceutical business and the public sector/education, whereas respondents in training or other sectors were situated in an intermediate position.

Figure 3 illustrates these discrepancies by presenting the mean index scores across several employment categories.

Pearson correlations were calculated to examine the links between the composite indices and the outcome variable (item 5.13). Table 6 shows that the indices had modest correlations. The strongest association was found between the Motivators Index and the likelihood of re-selecting a pharmacy (r = 0.40, p < 0.001), indicating that intrinsic factors, such as fulfilment, recognition, and career opportunities, were the most indicative of graduates’ long-term commitment. The Hygiene Index showed a strong correlation with the outcome (r = 0.33, p < 0.001). In contrast, the Pay-Equity Index had only a slight correlation with the outcome (r = 0.10, p < 0.05).

**TABLE 6:** Correlations among indices and willingness to re-choose pharmacy.

Variable	Hygiene	Motivators	Pay-equity	Outcome (5.13)
Hygiene	1	0.60**	0.28**	0.33**
Motivators	0.60**	1	0.25**	0.40**
Pay-equity	0.28**	0.25**	1	0.10*
Outcome (5.13)	0.33**	0.40**	0.10*	1

*p < .05, **p < .001.

**FIGURE 4:**
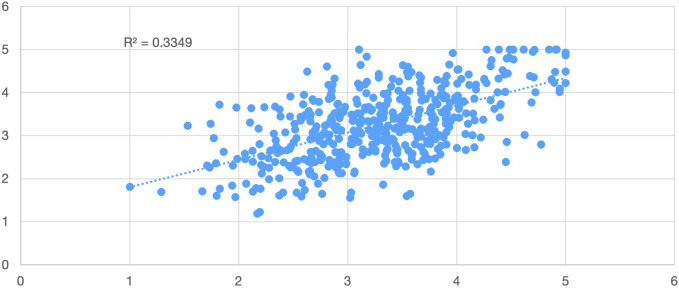
Correlation between Hygiene and Motivators indices (N = 473). Scatterplot with regression line (R^2^ = 0.39, p < 0.001).

In addition to their connections with the results, the indices demonstrated significant interrelationships. A robust positive connection was identified between Hygiene and Motivators (r = 0.62, p < 0.001). A linear regression revealed that Hygiene explained 39% of the variance in Motivators (R^2^ = 0.39), thus corroborating the convergent validity of the composite measures (Figure 4).

### Outcome on willingness to re-choose pharmacy

The outcome variable (item 5.13) revealed alarming levels of professional dissatisfaction. The response distribution was skewed towards negative evaluations: 45.9% of respondents selected “definitely not,” 29.3% “unlikely,” 7.0% “neutral,” 10.7% “probably,” and only 7.1% “definitely yes.” Consequently, nearly three out of four graduates (74.4%) showed little or no willingness to re-choose pharmacy as their degree. These results reinforce the item-level findings and highlight the wider disaffection within the profession.

**FIGURE 5:**
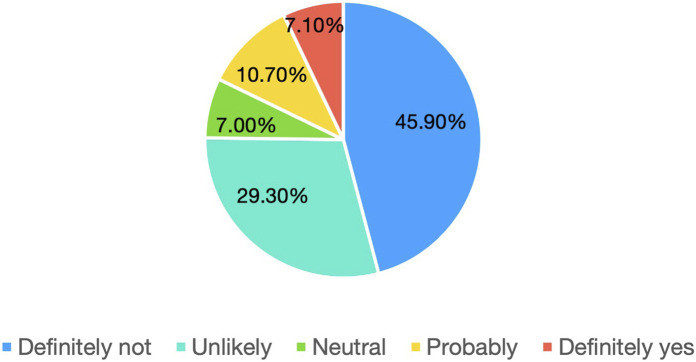
Willingness to re-choose pharmacy (item 5.13, n = 468).

Figure 5 displays the distribution of responses in a stacked bar chart, visually emphasising the dominance of negative categories. Notably, the high number of “definitely not” responses highlights the strong dissatisfaction among many graduates.

Pearson χ^2^ tests examined the relationships between the readiness to re-select a pharmacy (item 5.13) and demographic/professional characteristics. No notable correlations were detected for graduation year, university, or age group; however, mid-career graduates (ages 28–35) showed a tendency towards increased discontent. The employment sector revealed considerable variation: community pharmacists demonstrated a significantly lower likelihood to re-select pharmacy, whereas graduates from the industrial sector and public sector/education exhibited a greater inclination. The results are summarised in Table 7.

**TABLE 7:** Crosstabulations of willingness to re-choose pharmacy by demographic and professional variables.

Variable	χ^2^ (df)	p-value	Key pattern
Graduation year	60.26 (56)	0.324	No significant association across cohorts
University (graduation location)	52.91 (40)	0.083	Non-significant trend; no clear faculty differences
Age group	10.44 (12)	0.578	No significant differences across age brackets
Employment sector	27.85 (16)	0.033	Community pharmacy is the least likely to re-choose; industry/public is more positive

χ^2^ tests use the 5-category coded outcome (items 5.13 codes); cases with missing/”don’t know” responses are excluded (N = 468).

No significant relationships were identified with graduation year, university, or age group; yet, descriptive patterns suggested that mid-career graduates (ages 28–35) and the 2013 cohort demonstrated elevated dissatisfaction. On the other hand, the employment sector exhibited a significant disparity: respondents in community pharmacy demonstrated a substantially lower likelihood of selecting the same educational path. In contrast, those in the pharmaceutical industry and the public sector/education reflected a comparatively more positive perspective. The results support earlier findings from the ANOVA concerning sectoral differences in satisfaction indices.

## Discussion

### Summary of the results

Overall, the study’s results reveal moderate satisfaction with work conditions and collegiality, but deep dissatisfaction with salary and perceived equity. Community pharmacists reported the lowest scores across all domains. The low rate of career recommitment highlights the need to address systemic issues in the Romanian pharmacy profession.

Within Herzberg’s two-factor framework, salary is conceptualized primarily as a hygiene factor, meaning that its absence or inadequacy generates dissatisfaction but does not, by itself, create long-term engagement. The markedly low salary satisfaction observed in this study therefore explains the high levels of dissatisfaction. However, the low rate of career recommitment suggests that deficiencies extend beyond hygiene conditions. The comparatively weaker scores in motivator dimensions, particularly career prospects and recognition, may indicate a structural deficit in intrinsic drivers of professional engagement.

The Pay-Equity Index scored particularly low (mean = 1.86/5), with over 80% of respondents believing their income is significantly lower than that of physicians and dentists. Intrinsic motivators such as personal fulfilment and job challenge received slightly better ratings. Recognition and career advancement opportunities were still rated poorly. Community pharmacists consistently reported the lowest satisfaction across all indices, while those in the pharmaceutical industry and public sector/education showed relatively higher satisfaction.

Most strikingly, only 7% of respondents said they would “definitely” choose pharmacy again, while 46% said “definitely not,” and nearly three-quarters expressed little or no willingness to re-enter the profession. The strongest predictor of career commitment was the Motivators Index (r = 0.40), suggesting that intrinsic factors, such as fulfilment and recognition, play a key role in long-term engagement. The results reveal a troubling erosion of career commitment, highlighting the need for immediate action to improve pharmacists’ pay, enhance recognition, and establish clearer pathways for advancement to stabilise and strengthen the pharmacy workforce.

### Interpretation in the context of literature

This research provides one of the few quantitative evaluations of job satisfaction among Romanian pharmacy graduates, utilising Herzberg’s Two-Factor Theory and Adams’ Equity Theory within a multidimensional framework. Our findings illustrate a multifaceted scenario: although hygiene-related factors, including working conditions and collegial relationships, garnered moderate approval, satisfaction regarding salary and perceptions of equity were substantially deficient, with over 80% of participants deeming their income considerably lower than that of physicians and dentists. Motivational factors, such as personal fulfilment, career prospects, and professional growth, were assessed as moderate, suggesting they offer limited yet inadequate sources of intrinsic motivation. Reliability analyses validated that the three indices (Hygiene, Motivators, and Pay Equity) consistently emphasise aspects of satisfaction. In particular, the inclination to pursue pharmacy education again was minimal, with almost half of the participants indicating that they would “definitely not” opt for this career trajectory again. The results highlight a significant conflict between modest job security and collegiality, contrasting with persistent dissatisfaction over financial and career opportunities, which reflects global concerns about retention and workforce sustainability within the pharmacy profession.

The evaluation of hygiene and motivational characteristics reveals significant differences, including pharmacists’ professional satisfaction. Hygiene factors, including the workplace and job security, received a favourable assessment, consistent with the moderating influence of external conditions on employee dissatisfaction. Nevertheless, satisfaction with remuneration was the least favourable feature, underscoring that financial compensation remains a significant need in the Romanian setting. This finding aligns with global studies that demonstrate insufficient compensation routinely serves as a significant obstacle to personnel stability [27]. At the same time, a moderate evaluation of intrinsic motivators, such as professional growth, recognition, and personal fulfilment, revealed few but observable sources of internal motivation. Similar patterns have been noted in other pharmacy settings, where meaningful engagement and chances for professional development are essential components of a fulfilling career [28]. The observed balance (moderate fulfilment alongside weak recognition and career prospects) indicates that while Romanian pharmacists find meaning in their daily practice, structural constraints still hinder deeper engagement and sustained motivation.

Within Herzberg’s framework, this configuration suggests a dual mechanism. While inadequate hygiene factors such as salary generate dissatisfaction, the erosion of long-term engagement appears more strongly linked to deficits in motivators. The stronger correlation between the Motivators Index and willingness to re-choose pharmacy (r = 0.40) compared to Pay-Equity (r = 0.10) supports the interpretation that intrinsic drivers—recognition, career prospects, and professional growth—play a more decisive role in sustaining career commitment than financial dissatisfaction alone.

The Pay-Equity Index revealed the lowest levels of satisfaction, with the majority of respondents perceiving their income as substantially lower than that of doctors and dentists; this aligns with Adams’ Equity Theory, which highlights the centrality of perceived fairness in shaping job satisfaction and retention. Our findings are consistent with evidence from Japan, where career satisfaction among hospital pharmacists is strongly conditioned by career advancement opportunities and remuneration disparities [29]. Similarly, longitudinal analyses of pharmacy graduates in Ireland demonstrated lower satisfaction in community and hospital pharmacy roles compared to other sectors, with compensation emerging as an explanatory factor [30]. Additional insights from the United States show that alternative staffing models and workplace arrangements, such as hybrid work, can enhance satisfaction even when financial incentives remain constrained, illustrating the multidimensional nature of equity perceptions [31]. Taken together, these results suggest that financial inequity functions not only as a material constraint but also as a symbolic barrier, undermining the perceived value of pharmacists’ contributions and threatening the long-term sustainability of the workforce. Adams’ Equity Theory offers an explanatory perspective on these findings. The strong perception of income disparity relative to physicians, dentists, and other professionals with comparable educational investment suggests a perceived imbalance between input (years of study, professional responsibility) and output (financial reward). According to equity theory, such perceived under-reward may generate psychological tension and contribute to disengagement or reduced professional commitment, which may partially explain the low willingness to re-choose pharmacy observed in this sample.

The descriptive findings on monthly income and expectations for salary increases further illuminate the structural challenges faced by Romanian pharmacists. Reported income ranges clustered at modest levels, with half of respondents concentrated in lower categories, and expectations for financial improvement remained minimal. This configuration suggests a normalization of under-compensation, where professionals anticipate limited upward mobility. International evidence indicates that salary sufficiency and prospects for financial advancement are pivotal determinants of satisfaction and workforce stability. In Jordan, for instance, burnout among community pharmacists was strongly correlated with perceptions of inadequate salary and limited enjoyment of work [32]. Likewise, evidence from Pakistan suggests that work schedule flexibility can buffer stress; however, compensation remains a key predictor of well-being among hospital pharmacists [33]. In China, studies of community pharmacists have demonstrated that income, alongside continuing education and career prospects, plays a significant role in sustaining their capacity to provide pharmaceutical services [34]. Taken together, these findings underscore that financial constraints and restricted opportunities for advancement are not only demotivating but also erode the long-term viability of the pharmacy workforce.

Perhaps the most concerning outcome of this study is the low level of career commitment, as reflected in the fact that nearly half of the respondents stated they would “definitely not” choose pharmacy as their field of study again. This finding highlights a profound misalignment between professional expectations and lived realities, with implications that extend beyond individual dissatisfaction to the sustainability of the pharmacy workforce. International research points to similar patterns: younger cohorts of pharmacists often report higher levels of disillusionment, suggesting generational vulnerabilities in career attachment [35]. Broader evidence also associates dissatisfaction and regret with systemic issues such as heavy workloads, constrained autonomy, and unfulfilled professional identity [36]. Taken together, these results highlight that career disengagement is not only a Romanian phenomenon but also part of a wider international challenge, where inadequate compensation, limited progression, and structural barriers converge to weaken long-term professional attachment to the pharmacy profession.

The results of this study carry significant implications for both workforce policy and pharmaceutical education. Persistent dissatisfaction with salary and perceived inequity highlights the urgent need for remuneration reforms that better reflect pharmacists’ responsibilities and expertise. International evidence suggests that financial recognition, although not the sole driver of engagement, remains essential for workforce sustainability [37]. Beyond compensation, the moderate scores on professional development and recognition suggest that institutional support structures require strengthening. Tailored mentorship programs, leadership training, and structured opportunities for continuing education have been shown to mitigate burnout and improve career satisfaction in diverse pharmacy settings [38].

### Policy and educational implications

The study’s results highlight a critical need for policy-level interventions to address the widespread dissatisfaction among Romanian pharmacists. Chief among these is the need for remuneration reform—pharmacists consistently reported feeling underpaid, especially in comparison to physicians and dentists. This perceived inequity not only affects morale but also undermines the profession’s perceived value. Policymakers must prioritise fair compensation structures that reflect pharmacists’ responsibilities and contributions to healthcare, as financial recognition remains a foundational element of workforce sustainability. Beyond salary, the study emphasises the significance of institutional support mechanisms in enhancing job satisfaction and retention. These include structured mentorship programs, leadership development initiatives, and ongoing educational opportunities. Such initiatives have been shown in international contexts to reduce burnout and enhance long-term engagement. In Romania, where pharmacists face limited advancement opportunities and weak recognition, these support systems could play a transformative role in rebuilding trust and motivation within the profession.

From an educational perspective, the low level of career commitment among graduates indicates a disconnect between academic training and real-world professional experiences. Pharmacy curricula must evolve to better reflect the diverse and changing roles pharmacists can occupy, including opportunities in industry, research, and public health. Universities should also focus on developing transferable skills and career planning resources that prepare students for a broader range of career paths beyond traditional community or hospital settings. Ultimately, collaboration among educational institutions, professional bodies, and policymakers is crucial for implementing these changes effectively. By aligning educational content with labour market demands and ensuring that pharmacists are adequately supported and compensated, Romania can begin to reverse the current trend of professional disengagement. The findings suggest that recommended reforms should directly address the empirical deficits identified in this study. The very low Pay-Equity Index (M = 1.86) indicates the need for remuneration adjustments and improved transparency. The modest scores for recognition (M = 2.77) and career prospects (M = 2.95), together with the strong association between the Motivators Index and career recommitment (r = 0.40), highlight the importance of structured career pathways and formal recognition systems.

### Future research directions

Future research should pursue three main directions. First, longitudinal and mixed-methods designs are required to capture dynamic changes in satisfaction and career trajectories, particularly in the context of evolving healthcare systems. Second, comparative studies across Eastern Europe would help disentangle national versus regional drivers of dissatisfaction and commitment. Third, intervention-based research (testing the effects of mentorship, leadership training, salary reform, or continuing education initiatives) would provide actionable evidence to guide both policy and institutional reforms. Addressing these gaps will be essential for developing a comprehensive understanding of professional satisfaction and ensuring the sustainability of the pharmacy workforce.

### Limitations of the study

Several limitations should be acknowledged when interpreting these findings. First, the cross-sectional design prevents any inference about causality between satisfaction dimensions and career commitment. Longitudinal studies are necessary to evaluate how satisfaction evolves over time and whether dissatisfaction early in a career predicts attrition or sectoral mobility. Second, the reliance on self-reported measures introduces the risk of social desirability bias and recall bias, which can potentially lead to underestimation or overestimation of satisfaction levels. Third, although the sample was large and included graduates from all primary Romanian pharmacy schools, it was not random and may have overrepresented individuals motivated to share their experiences, particularly those with strong negative views. The strong gender imbalance in the sample, reflecting broader trends in the profession, also limits the generalizability of subgroup analyses. Finally, the focus on quantitative indices, while valuable for comparability, may overlook nuanced qualitative aspects of professional identity, resilience, and coping strategies.

The study does not fully explore how external events (e.g., economic shifts, healthcare reforms, or the COVID-19 pandemic) during the 2009–2023 period may have influenced responses. Graduates from different years may have experienced very different labour market conditions, which could affect their satisfaction and career outlook.

Although the study compares satisfaction across employment sectors, it does not account for regional disparities within Romania or distinguish between urban and rural work environments. These contextual factors could have a meaningful impact on pharmacists’ job satisfaction and career choices, yet they remain unexplored in the current analysis.

## Conclusion

This study provides new insights into the professional satisfaction of Romanian pharmacy graduates, revealing moderate assessments of workplace conditions and collegiality, but consistently low ratings for salary, fairness, and career advancement opportunities.

At the same time, the large number of graduates unwilling to rechoose pharmacy highlights significant challenges in maintaining long-term career engagement.

This study highlights substantial dissatisfaction with pay equity and moderate but commitment-relevant deficits in intrinsic motivators among Romanian pharmacy graduates. The very low Pay-Equity Index and the weaker scores for recognition and career prospects, together with their association with career recommitment, suggest that both financial and structural reforms are necessary to restore long-term professional engagement.

As concrete next steps, national professional bodies could implement transparent remuneration review mechanisms and develop structured, competency-based career progression frameworks within community pharmacy. Such targeted interventions may represent measurable strategies to enhance workforce stability and retention.

## Data Availability

The raw data supporting the conclusions of this article will be made available by the authors, without undue reservation.
